# Decreased complications but a distinctive fixation loosening mechanism of fully threaded headless cannulated screw fixation for femoral neck fractures in young adults

**DOI:** 10.1186/s13018-021-02335-3

**Published:** 2021-03-30

**Authors:** Hui Sun, Lin-Yuan Shu, Matthew C. Sherrier, Yi Zhu, Jing-Wen Liu, Wei Zhang

**Affiliations:** 1 Department of Orthopaedic Surgery, Shanghai Jiao Tong University Affiliated Sixth People’s Hospital, 600 YiShan Road, Shanghai, 200233 China; 2 Department of Emergency Medicine, Shanghai Jiao Tong University, Affiliated Sixth People’s Hospital, 600 YiShan Road, Shanghai, 200233 China; 3 Department of Physical Medicine and Rehabilitation, University of Pittsburgh Medical Center, Pittsburgh, PA 15219 USA

**Keywords:** Femoral neck fracture, Internal fixation, Fully threaded headless cannulated screws, Complication, Fixation failure

## Abstract

**Background:**

Despite being a commonly encountered injury in orthopedic practice, controversy surrounds the methods of optimal internal fixation for femoral neck fractures (FNF) in young patients. The objective of the present study is to compare complication rates and failure mechanisms for surgical fixation of FNF using fully threaded headless cannulated screws (FTHCS) versus partial threaded cannulated screws (PTS) in young adults.

**Methods:**

A total of 75 patients (18–65 years old) with FNF were prospectively treated with close reduction and internal fixation using three parallel FTHCS and compared to a historical control case-matched group (75 patients) with FNF treated by PTS fixation. After 2 years follow-up, rates of fixation failure (including varus collapse, fracture displacement, and femoral neck shortening), nonunion, and avascular necrosis of the femoral head (ANFH) were compared between the two cohorts. The demographic, follow-up information, and radiological images were assessed by independent blinded investigators.

**Results:**

Patient demographics and fracture patterns were similar in the two patient groups. The overall fixation failure rates were 8% (6/75) in the FTHCS cohort, which was significantly lower than the 25.3% (19/75) seen in the PTS group. Rates of nonunion and ANFH were significantly lower in the FTHCS group when compared to the PTS control group. When stratified by injury severity (high-energy vs. low-energy fractures), the rate of fixation failure was significant lower with the use of FTHCS when compared with PTS for high-energy fractures while there was no difference in the rates of nonunion or ANFH for high or low-energy fracture patterns. Unique to the FTHCS cohort was an atypical screw migration pattern with varus collapse (6/75, 8%).

**Conclusions:**

The results show that FTHCS fixation could significantly reduce the complication rate of young patients with FNF, especially in high-energy fracture patterns (Garden III–IV, Pauwels III, or vertical of the neck axis (VN) angle ≥ 15°). There was also confirmation that the modes of fixation loosening in the FTCHS group, including screw “medial migration” and superior cutout, were different from the screw withdrawal pattern seen in the PTS cohort.

**Trial registration:**

The study was retrospectively registered at www.Chictr.org.cn (ChiCTR-IPR-1900025851) on September 11, 2019.

## Background

Femoral neck fractures (FNF) are a commonly encountered injury in orthopedic practice that are associated with substantial morbidity, mortality, and costs [1]. The high proportion of fixation failures resulting in reoperation has generated controversy about the most appropriate surgical treatment for FNF. FNF in young adult patients are typically associated with high-energy trauma mechanisms and displaced fracture patterns, resulting in a biomechanically disadvantageous environment for fracture healing [2, 3]. In high-energy fracture patterns, fixation failure, malunion, and avascular necrosis of the femoral head (ANFH) result from disruption of the vascular supply, biomechanical instability, or both [4]. This is in contrast to low-energy fracture patterns, which are frequently associated with femoral neck shortening, due to bone resorption and sliding fixation during remodeling [5]. Additionally, younger patients are more likely to have higher post-operative functional demands for work or recreational activities. As such, treatment of FNF in young patients presents a clinical challenge when choosing the optimum fixation approach and is associated with significant complications.

The principles of stable fixation for FNF healing include fracture compression, resistance to shear and rotational forces, and achievement of anatomic reduction [6, 7]. Established fixation methods for FNF include multiple cancellous screws, fixed-angle dynamic implants, and fixed-angle length-stable constructs [3, 8, 9]. Partial threaded cannulated screws (PTS) are a mainstay of treatment and have the advantages of minimal tissue invasiveness and intraoperative blood loss, decreased hospital stay and operation time while also preserving the native blood supply [10]. PTS fixation compresses the fracture to obtain primary stability and allows the fracture fragments to slide along the implant to enhance secondary stability when subjected to a weight-bearing axial load. Despite the aforementioned benefits, femoral neck shortening [11, 12] or fixation failure [9] has been reported to occur with PTS. Although a large amount of biomechanical and clinical studies have evaluated various fixation options, the optimal fixation construct to allow for healing and prevention of complications after FNF is still unknown [3].

In recent years, the use of fully threaded cannulated screws for FNF fixation has gained attention. When compared to PTS, fully threaded headless cannulated screws (FTHCS) have been shown to minimize femoral neck shortening [13, 14]. Recent clinical studies have evaluated the potential of FTHCS to reduce other complications of FNF fixation, such as fixation failure and nonunion [10, 15, 16]. Unfortunately, a limited number of these clinical studies have led to uncertain conclusions due to small sample sizes and varying demographic patterns [10, 13–16]. In order to gain clarity, the purpose of the present study is to examine the complication rates of FTHCS compared to PTS for surgical fixation of FNF in a non-geriatric population using a larger sample size than previous studies. We hypothesize that the utilization of FTHCS will result in fewer fixation failures (including varus collapse, fracture displacement, and femoral neck shortening > 10 mm), nonunion, and ANFH when compared to PTS fixation.

## Methods

We completed a prospective cohort study with historical controls to compare the 2-year postoperative outcomes of patients under the age of 65 undergoing FNF fixation (OTA/AO classification 31-B [17]) in a level I trauma center using three parallel FTHCS versus those who received three parallel PTS. Exclusion criteria for the prospective FTHCS cohort included patients with an immature skeletal system (age ≤ 18 years), pathological and old fractures, previous hip surgery, deformity, or dysplasia of the ipsilateral hip or femur. Additionally, patients who received an open reduction, those with multiple injuries (injury severity scale, ISS > 16), or accompanying fractures of the ipsilateral lower extremity, femoral head fracture, pelvic or acetabular fracture, which might influence the process of rehabilitation and weight-bearing were also excluded.

We compared the prospective FTHCS cohort to a matched historic cohort that received the traditional common practice of three parallel PTS fixation for FNF. Matching criteria included sex, age (18–65 years), body mass index (BMI) within 3 kg/m^2^, comorbidities, injury laterality, and fracture classification. The primary exclusion criteria were identical to the FTHCS population. Additionally, patients without clinical assessment during follow-up, perioperative and follow-up radiographs, or lack of follow-up were excluded from the study. A complete medical record was available in the electronic medical record, including comorbidities, radiological images, operation details, and follow-up outcomes. These records provided valid information for the retrospective analysis.

The study was conducted in compliance with the principle of the Declaration of Helsinki, approved by the institutional review board of our center, and registered at www.Chictr.org.cn (ChiCTR-IPR-1900025851). Each patient in the prospective group who planned to follow-up at least 24 months signed an informed consent form agreeing to participate in this study.

### Fracture management and postoperative management

In the Emergency Department, all patients underwent a standard radiological protocol of x-rays, including anteroposterior (AP) view of the pelvis and lateral view of the injured hip, as well as computed tomography (CT) scans and image reconstruction. Preliminary management included skin traction or bony skeletal traction to reduce and maintain limb alignment. On admission, demographics and mechanism of injury were recorded.

All surgeries in this study were performed by the team of authors (H.S. and W.Z.), orthopedic traumatologists with at least 10 years of experience. The patients in both groups were given either general or regional anesthesia and positioned supine on a fracture table. Limb length was restored intraoperatively by gentle longitudinal traction under an image intensifier. Restoration of rotational malalignment was accomplished via internal or external rotation of the extremity. In each operation, the expectation of acceptable rotational reduction was slight valgus or anatomic reduction on the AP view (neck-shaft angle between 130 and 150°) and no posterior collapse or anterior angulation (less than 15° anteversion) on the real femoral lateral view [18, 19]. As a result of the lack in general consensus on grading the quality of FNF reduction, fracture reduction was graded on the amount of displacement and the degree of residual angulation, matching published criteria [7]. An excellent reduction is considered less than 2 mm of displacement and 5° of angulation in any plane; good reduction is 2–5 mm of displacement and/or 5°–10° of angulation; fair reduction is 5–10 mm of displacement and/or 10°–20° of angulation. Displacement exceeding 10 mm or an angulation of 20° is considered poor. After reduction, a guidewire was inserted up to the subchondral bone of the femoral head and was then measured and drilled. Three absolute FTHCS (Acutrak 6/7, Acumed, Hillsboro, OR, US) or two FTHCS with one PTS were implanted in parallel. If no obvious comminution on the neck cortex was seen, a PTS could be implanted first to compress the fracture site prior to FTHCS implantation. Additionally, prior to FTHCS implantation, the lateral cortex of the proximal femur was tapped to reduce twisting forces. PTS (Asnis III 6.5 mm, Stryker, Howmedic, Mahwah, NJ, US) were implanted via manufacturer instruction. Either regular or inverse triangle configuration was determined by the surgeons according to their own experience, because there is no literature available for reference about which configuration is more effective for FTHCS fixation.

A standard postoperative rehabilitation protocol was followed regardless of the fixation technique performed. All patients were non-weight bearing for at least 8 weeks after surgery. When radiographic and clinical healing appeared to be progressing toward union, weight bearing was advanced slowly from toe touch to partial weight bearing as tolerated over the subsequent 6 weeks, at the discretion of the treating surgeon.

All patients had routine follow-up. Physical examination was performed and standard radiographs were obtained at each follow-up visit. Postoperative CT or magnetic resonance imaging (MRI) was utilized at the discretion of the treating surgeon to evaluate for nonunion or ANFH. The time to radiographic union, Harris Hip Score (HHS), and any complications observed at any time during follow-up were recorded.

### Fracture classification

The Garden [18], modified Pauwels [20], and vertical of the neck axis (VN) angle [21] classifications of FNF in each patient were assessed by two independent investigators (L-Y. S. and J-W. L.). Disagreements were settled by a third, trauma-trained orthopedic surgeon (Y.Z.). All three investigators were blinded to treatment. The radiological images were obtained using picture archiving and communication system workstations. Measurements were performed using Kingstar Winning TV view software (Shanghai Kingstar Winning Medical Information Technology Co. Ltd., Shanghai, China). Due to poor intra-observer and inter-observer reliability by using the various classifications [21], both modified Pauwels classification and VN angle were applied. For the purpose of research, low-energy fractures were just defined as Garden I–II, Pauwels I–II, or VN < 15° patterns and high-energy fractures were defined as Garden III–IV, Pauwels III, or VN ≥ 15° patterns in this study [20, 21].

### Outcome measures

**Fig. 1:**
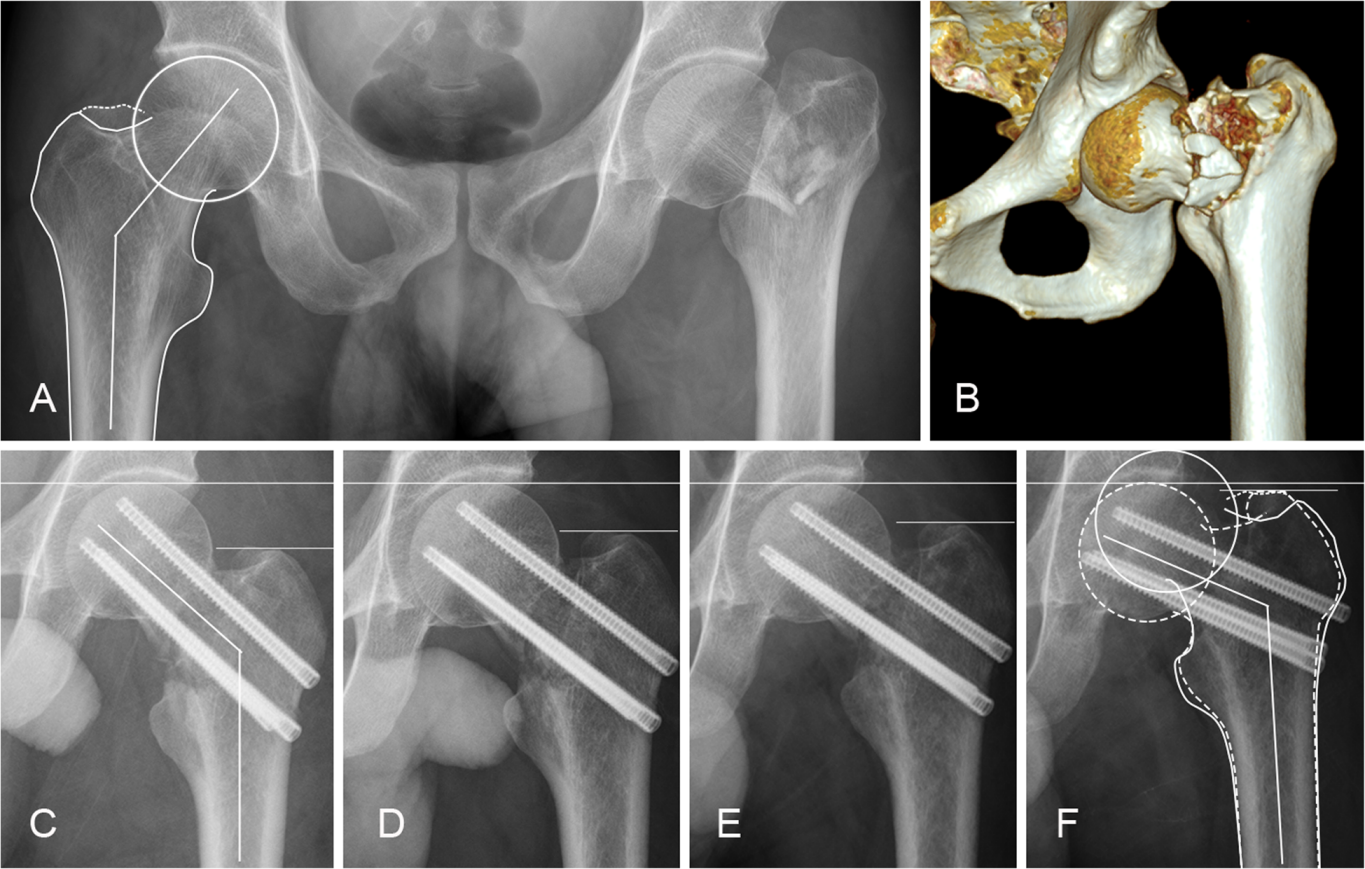
FTHCS fixation for FNF in a 40-year-old complicated by femoral neck shortening. The pelvic AP radiograph (**a**) and CT scan 3-D reconstruction images (**b**) showed the right hip with a comminuted femoral neck fracture. **a** The uninjured hip was outlined and the angle between the axis of the head and shaft (neck-shaft angle) was labeled. Immediate postoperative radiographs (**c**) showed near anatomical reduction with corrected neck-shaft angle. One-month (**d**) and 3-month (**e**) postoperative radiographs showed stability of the fixation. Unfortunately, 2-year radiographs (**f**) demonstrated fracture union with significant femoral neck shortening and varus displacement. The outline of the uninjured hip (solid line) overlapped on the fracture side (dotted line) is provided for comparison. From (**c**) to (**f**), two horizontal lines were drawn on each radiograph, one from the top of the femoral head and another from the tip of the greater trochanter. The difference in the measurement between these two horizontal lines revealed the amount of head collapse

Our primary outcome was fixation failure, defined as varus collapse (> 10°), fracture displacement (> 5 mm), or femoral neck shortening (> 10 mm vertically). To evaluate fixation failure, the immediate postoperative radiographs were compared with follow-up radiographs. For varus collapse, the change in neck-shaft angle between the postoperative and follow-up radiographs was measured on pelvic AP radiographs (Fig. 1a, c, f). Femoral neck shortening was evaluated on pelvic AP view of postoperative and follow-up radiographs. On these images, two horizontal lines were drawn perpendicular to the femoral anatomical axis from the top of the femoral head and the tip of the greater trochanter on bilateral hips. Vertical femoral neck shortening was defined as the bilateral difference between the two lines (Fig. 1c–f) [14]. Although other methods of measuring shortening using the contralateral hip have been validated in prospective case series [11, 15], they are not suitable for historical control cases where leg rotation could not be standardized.

Secondary outcomes included fixation loosening, nonunion, and ANFH. Fixation loosening was identified if there was any screw penetration or withdrawal on radiographs. Screw withdrawal can manifest clinically as soft tissue irritation in the greater trochanter region. If the fracture line was grossly visible at 6 months postoperatively, a fracture nonunion was considered to be present [22]. The radiographic criteria of Ficat and Arlet were used to diagnose ANFH [23].

### Statistical methods

Based on previous results from the treatment of vertical FNF [10], the rate of fixation failure in the group treated with PTS alone was 41.9%, while fixation failure rate in the group treated with FTHCS was 14.3%. A two-tailed test was performed with an α level of 5% (α = 0.05) and power of 80% (β = 0.20) to determine that a sample size of 43 would be required in each group to gain significant results. Considering a certain proportion of non-vertical fractures (40%) in adult patients under the age of 65, a rate of lost to follow-up of 10%, and the rate of open reduction of 10%, we decided to enroll 100 patients in the prospective FTHCS fixation group.

The sample size was calculated using G*Power 3.1.9.3 software (Institute for experimental psychology in Dusseldorf, Germany). Statistical analyses were performed by an independent statistician blinded to clinical outcomes using SPSS 19.0 (SPSS, Inc., Chicago, IL, US). Continuous variable were presented as mean ± SD, and tested by Student’s *t* test. Categorical variables were shown as number and percentages (%) and tested by the chi-squared test. Fisher’s exact test was implemented when 20% of the cells had expected values less than 5.

After controlling for important confounders in the cohorts, a multivariable logistic regression model was used to determine the independent risks of complication associated with fixation failure, nonunion, and ANFH. Logistic regression analysis results were presented as odds ratios (OR) with 95% confidence intervals (95% CI). Stratified analysis using the same regression models was then performed to characterize differences in the strength of the fixation methods across the fracture classification (injury severity). A value of *p* < 0.05 indicated statistical significance.

## Results

**Fig. 2:**
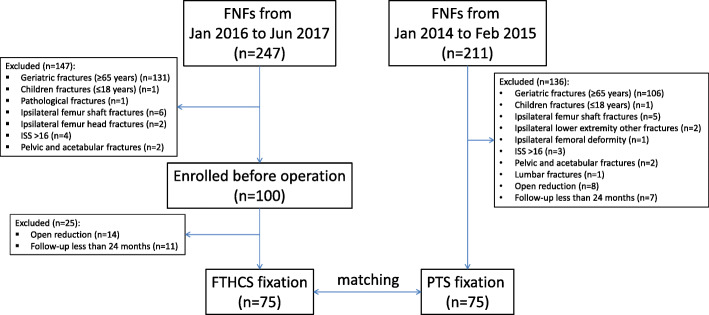
Flow chart showing the prospective participants matched with a historical cohort in the study

From January 2016 to June 2017, a total of 247 patients with FNF were screened. One hundred patients who fulfilled all inclusion and exclusion criteria were prospectively enrolled for FNF fixation with FTHCS. Subsequently, 14 patients were excluded due to open reduction and 11 patients were lost to follow-up due to voluntary withdrawal (2), moved (8), or foreign domicile (1). Seventy-five patients were followed up at a minimum of 24 months postoperatively, resulting in a rate of follow-up of 87.2% (75/86). Seventy-five patients with FNF from 211 patients (treated from January 2014 to February 2015) fixed by three PTS met the conditions to be retrospectively matched (Fig. 2). There were no statistically significant differences in the demographics, comorbidities, or fracture classifications between the FTHCS and PTS cohorts (Table 1). Additionally, the operation time, fracture reduction quality, and screw configuration were similar for both groups, except the blood loss during operation, which was greater in the FTHCS cohort (*p* < 0.05) (Table 2).

**Table 1:** Demographics and fracture characteristics

Variables	FTHCS (*n* = 75)	PTS (*n* = 75)	*p* value
Age (years)	48.76 ± 9.62	49.88 ± 10.90	0.506
Male gender (%)	38 (50.7%)	34 (45.3%)	0.513
Right side (%)	28 (37.3%)	35 (46.7%)	0.247
Comorbidities			
BMI (kg/m^2^)	24.75 ± 3.12	23.91 ± 3.39	0.119
Smoker (%)	22 (29.3%)	26 (34.7%)	0.484
Alcohol abuse (%)	8 (10.7%)	13 (17.3%)	0.239
Diabetes (%)	21 (28.0%)	18 (24.0%)	0.577
Cause of injury (%)			
Traffic vehicle accident	37 (49.3%)	40 (53.3%)	0.917*
Fall	14 (18.7%)	13 (17.3%)	
Pedestrian/ bicyclist struck	20 (26.7%)	17 (22.7%)	
Sport	4 (5.3%)	5 (6.7%)	
Fracture classification Garden classification (%)			
Garden III–IV	48 (64.0%)	46 (61.3%)	0.736
Pauwels classification (%)			
Pauwels III	47 (62.7%)	45 (60.0%)	0.737
VN classification (%)			
VN angle ≥ 15°	44 (58.7%)	39 (52.0%)	0.412
Fracture morphology (%)			
Posterior cortex communication	30 (40.0%)	26 (34.7%)	0.500

*FTHCS* Fully threaded headless cannulated screw fixation, *PTS* Partial threaded cannulated screw fixation, *BMI* Body mass index, *VN* Vertical of the neck axis

*Fisher’s exact test

**Table 2:** Operation information

Variables	FTHCS (*n* = 75)	PTS (*n* = 75)	*p* value
Operating time (minutes)	46.60 ± 13.08	45.92 ± 12.33	0.744
Blood loss (ml)	109.33 ± 50.41	89.20 ± 47.34	*0.013*
Quality of femoral neck reduction (%)			
Excellent	48 (64.0%)	50 (66.7%)	0.877*
Good	24 (32.0%)	23 (30.7%)	
Fair	3 (4.0%)	2 (2.7%)	
Poor	0 (0.0%)	0 (0.0%)	
Configuration of screws (%)			
Regular triangle	51 (68.0%)	56 (74.7%)	0.367

*FTHCS* Fully threaded headless cannulated screw fixation, *PTS* Partial threaded cannulated screw fixation

*Fisher’s exact test

Our primary outcome, the fixation failure rate, was significantly lower in the FTHCS cohort when compared to the PTS cohort (*p* < 0.01). Additionally, there were significantly lower rates of nonunion and femoral neck shortening < 10 mm along with decreased time to radiographic union in the FTHCS cohort when compared to the PTS cohort (*p* < 0.05). However, there was no statistically significant difference in the ANFH rate (*p* = 0.754). From a functional standpoint, although the HHS was significantly higher in the FTHCS cohort at the end of follow-up (*p* < 0.01), it did not reach the minimal clinically important improvement (Table 3) [24].

**Table 3:** Outcomes and follow-up data

Variables	FTHCS (*n* = 75)	PTS (*n* = 75)	*p* value
Follow-up duration (months)	26.96 ± 5.45	27.81 ± 5.50	0.342
Time to radiographic union (weeks)	16.64 ± 4.16	21.20 ± 10.13	*0.000*
HHS	89.96 ± 8.64	85.51 ± 9.93	*0.004*
Hardware removal (%)	62 (82.7%)	66 (88.0%)	0.356
Complications (%)			
Fixation failure	6(8.0%)	19(25.3%)	*0.004*
Nonunion	5 (6.7%)	13 (17.3%)	*0.044*
ANFH	5 (6.7%)	6 (8.0%)	0.754
Femoral neck shortening (< 10 mm)	8 (10.7%)	18 (24.0%)	*0.031*
Fixation loosening (%)			
Lateral withdrawal	16 (21.3%)	42 (56.0%)	*0.000*
Medial migration	6 (8.0%)	0 (0.0%)	0.028*

*FTHCS* Fully threaded headless cannulated screw fixation, *PTS* Partial threaded cannulated screw fixation, *HHS* Harris Hip score, *ANFH* Avascular necrosis of the femoral head

*Fisher’s exact test

**Fig. 3:**
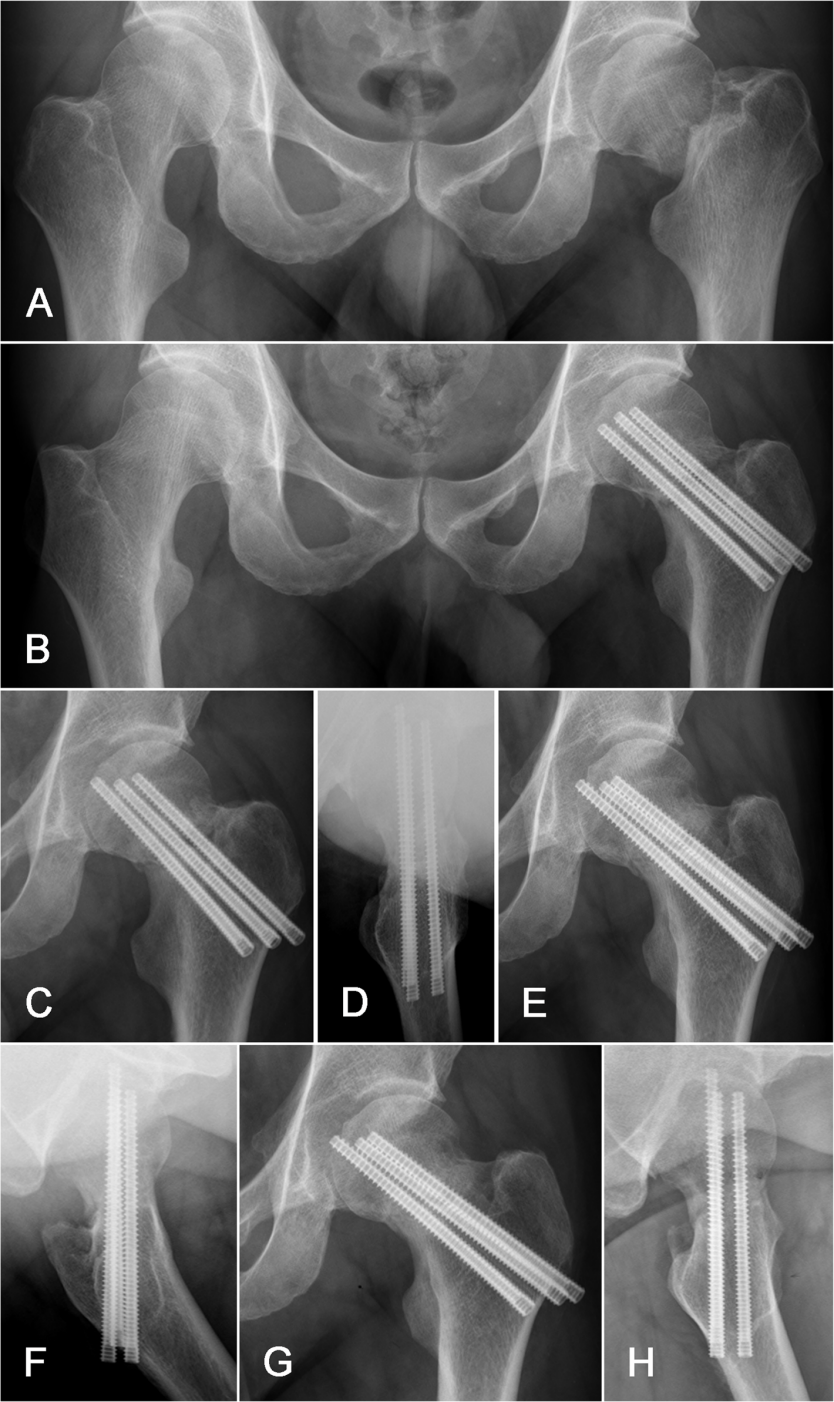
FTHCS fixation for FNF in a 43-year-old male complicated by medial screw migration. **a** AP radiograph of the pelvis including both hip joints revealed a Garden type IV, Pauwels type II left femoral neck fracture with VN angle < 15°. **b** Immediate postoperative radiographs showed acceptable reduction and three FTHCS. AP and lateral radiographs 3 months postoperatively (**c**, **d**) demonstrated migration of the distal screw beneath the femoral head subchondral bone in the direction of the acetabulum with slight fracture displacement. Radiographs 4 months postoperatively (**e**, **f**) exhibited further displacement of the distal screw tip, penetrating the subchondral bone. Six-month postoperative radiographs (**g**, **h**) revealed fracture union with no further shift of the displaced screw and the patient was without functional limitations

With regards to fixation loosening, there were significantly higher rates of lateral withdrawal represented by greater trochanter region soft tissue irritation in the PTS cohort when compared to the FTHCS cohort (*p* < 0.01). Interestingly, a distinct fixation loosening mechanism was observed in six patients in the FTHCS group (Table 3). In four cases, one of the three screws migrated medially, resulting from lateral migration of the femoral head and subsequent medial penetration of the miserable screw (Fig. 3). Additionally, two cases suffered from one screw superior cutout resulting from varus collapse. The medial screw migrations all occurred in the distal screw of the inverted triangle configuration and the superior cutout appeared in the proximal anterior screw. All cases suffered from posterior cortex communication and received an inverted triangle configuration. There were no such complications in the PTS group compared to an 8.0% (6/75) rate in the FTHCS group (*p* < 0.05). Of note, reduction quality was rated as excellent or good for all aforementioned fixation loosening cases (Table 2).

Given the distinct fixation failure mechanisms of low-energy and high-energy fracture patterns and that high-energy fractures are more common in young patients [4, 5], we next stratified complication outcomes based on injury severity. In high-energy fractures, the FTHCS fixation group exhibited significantly less fixation failure when compared to the PTS cohort and there was no significant difference in the rates of nonunion or ANFH between the fixation techniques (Table 4). There was no significant difference in the rate of fixation failure between FTHCS and PTS fixation in the low-energy fracture group. Furthermore, there was no significant difference in nonunion or ANFH between the fixation techniques in the low-energy fracture group. However, PTS fixation showed significantly higher rates of lateral screw withdrawal and femoral neck shortening < 10 mm than the FTHCS group in high- and low-energy fractures.

**Table 4:** Fixation complication outcomes comparison stratified by severity classifications

Severity	Fixation methods	Lateral withdrawal (%)	Femoral neck shortening (< 10 mm) (%)	Fixation failure (%)	Nonunion (%)	ANFH (%)
Garden I–II	FTHCS (*n* = 27)	2 (7.4%)	2 (7.4%)	1 (3.7%)	0 (0.0%)	2 (7.4%)
	PTS (*n* = 29)	11 (37.9%)	9 (31.0%)	6 (20.7%)	3 (10.3%)	1 (3.4%)
	*p* value	0.010*	0.042*	0.103*	0.237*	0.605*
Garden III–IV	FTHCS (*n* = 48)	14 (29.2%)	6 (12.5%)	5 (10.4%)	5 (10.4%)	3 (6.3%)
	PTS (*n* = 46)	31 (67.4%)	9 (19.6%)	13 (28.3%)	10 (21.7%)	5 (10.9%)
	*p* value	*0.000*	0.350	*0.028*	0.134	0.481*
Pauwels I–II	FTHCS (*n* = 28)	3 (10.7%)	3 (10.7%)	1 (3.6%)	0 (0.0%)	2 (7.1%)
	PTS (*n* = 30)	11 (36.7%)	11 (36.7%)	4 (13.3%)	3 (10.0%)	1 (3.3%)
	*p* value	0.031*	0.031*	0.354*	0.238*	0.605*
Pauwels III	FTHCS (*n* = 47)	13 (27.7%)	5 (10.6%)	5 (10.6%)	5 (10.6%)	3 (6.4%)
	PTS (*n* = 45)	31 (68.9%)	7 (15.6%)	15 (33.3%)	10 (22.2%)	5 (11.1%)
	*p* value	*0.000*	0.484	0.008*	0.133	0.481*
VN angle < 15°	FTHCS (*n* = 31)	4 (12.9%)	2 (6.5%)	1 (3.2%)	1 (3.2%)	2 (6.5%)
	PTS (*n* = 36)	15 (41.7%)	12 (33.3%)	7 (19.4%)	7 (19.4%)	2 (5.6%)
	*p* value	0.014*	0.008*	0.060*	0.060*	1.000*
VN angle ≥ 15°	FTHCS (*n*=44)	12 (27.3%)	6 (13.6%)	5 (11.4%)	4 (9.1%)	3 (6.8%)
	PTS (*n* = 39)	27 (69.2%)	6 (15.4%)	12 (30.8%)	6 (15.4%)	4 (10.3%)
	*p* value	*0.000*	0.821	*0.029*	0.504*	0.701*

*FTHCS* Fully threaded headless cannulated screw, *PTS* Partial threaded cannulated screw, *VN* Vertical of the neck axis

* Fisher’s exact test

Binary logistic regression revealed that screw fixation method was an independent risk factor for complication of fixation failure and femoral neck shortening < 10 mm (Table 5). FTHCS fixation was associated with a 74% lower risk of fixation failure (OR, 0.26; 95% CI, 0.10–0.69; *p* = 0.007) and a 62% lower risk of femoral neck shortening < 10 mm (OR, 0.38; 95% CI, 0.15–0.93; *p* = 0.035), as compared with PTS fixation. In a subgroup analysis, fixation method remained an independent risk factor for fixation failure in high-energy fractures (Garden III–IV: OR, 0.30; 95% CI, 0.10–0.91; Pauwels III: OR, 0.19; 95% CI, 0.06–0.62; VN ≥ 15°: OR, 0.29; 95% CI, 0.09–0.91; all *p* < 0.05) rather than in low-energy fractures. However, fixation method was an independent risk factor for femoral neck shortening < 10 mm in low-energy fractures (Garden I–II: OR, 0.18; 95% CI, 0.03–0.92; Pauwels I–II: OR, 0.21; 95% CI, 0.05–0.85; VN < 15°: OR, 0.14; 95% CI, 0.03–0.68; all *p* < 0.05). Again, fixation method was not a significant risk factor for rates of nonunion and ANFH in either the overall or severity-stratified groups (Table 5).

**Table 5:** Binary logistic regression models

Variables	Femoral neck shortening (< 10 mm) (FTHCS versus PTS)			Fixation failure (FTHCS versus PTS)				Nonunion (FTHCS versus PTS)			ANFH (FTHCS versus PTS)		
	OR	95% CI	*p* value	OR		95% CI	*p* value	OR	95% CI	*p* value	OR	95% CI	*p* value
Total	0.38	0.15–0.93	*0.035*	0.26		0.10–0.69	*0.007*	0.34	0.12–1.01	0.052	0.82	0.24–2.82	0.754
Garden classification													
I–II	0.18	0.03–0.92	*0.039*	0.15		0.02–1.32	0.087	–	–	0.998	2.24	0.19–26.23	0.521
III–IV	0.59	0.19–1.81	0.353	0.30		0.10–0.91	*0.034*	0.42	0.13–1.34	0.142	0.55	0.12–2.43	0.428
Pauwels classification													
I–II	0.21	0.05–0.85	*0.029*	0.50		0.08–2.97	0.446	–	–	0.998	2.23	0.19–26.06	0.522
III	0.65	0.19–2.21	0.486	0.19		0.06–0.62	*0.006*	0.42	0.13–1.33	0.140	0.55	0.12–2.43	0.427
VN angle													
< 15°	0.14	0.03–0.68	*0.015*		0.14	0.02–1.19	0.072	0.14	0.02–1.19	0.072	1.17	0.16–8.85	0.877
≥ 15°	0.87	0.26–2.95	0.821		0.29	0.09–0.91	*0.035*	0.55	0.14–2.11	0.384	0.64	0.13–3.06	0.640

*FTHCS* Fully threaded headless cannulated screw, *PTS* Partial threaded cannulated screw, *VN* Vertical of the neck axis, *ANFH* Avascular necrosis of femoral head

## Discussion

Based on previous preliminary results of biomechanical testing [10, 25] and a prospective clinical study with a relatively small sample size [10], FTHCS appears to have an advantage in the fixation of FNF in comparison to the traditional PTS fixation, especially in high-energy fractures [25]. The present study is the first to assess, in a large sample size, the clinical effectiveness of FTHCS compared to PTS in young patients with the full spectrum of high- and low-energy FNF patterns. The present findings support our hypothesis and suggest that the use of FTHCS for the treatment of FNF in young adult patients is associated with a reduced rate of fixation failure when compared with PTS, particularly in high-energy fracture patterns. We also found no significant difference in the rates of nonunion or ANFH between the FTCHS and PTS cohorts.

Controversy surrounds the methods of internal fixation for FNF in young patients. Studies on the clinical utility of multiple FTHCS fixation for FNF have yielded conflicting results. A prospective comparative study by Zhang et al. focusing on young patients (average age, 50 years) with VN > 20° FNF suggested that fixation with FTHCS resulted in lower fixation failure rate than PTS (14.3% vs. 41.9%) [10]. Meanwhile, others have found that, in comparison with FTHCS, PTS provides a shorter union time and lower complication rate (9.1% vs. 36.3%) [16]. Of note, the population in this study was relatively young (average age, 44 years) and fracture severity pattern was equally distributed [16]. Chiang et al. concluded that the FTHCS cannot prevent femoral neck shortening and varus collapse after fracture fixation and demonstrated similar complication rates (nonunion and ANFH) between FTHCS and PTS (17.6% vs. 21.2%) [15]. However, the results were mainly based on geriatric patients (average age, 71.7 years) and low-energy fracture patterns (Pauwels I–II 90%) [15]. The opposing conclusions of these publications are in part due to small sample size and lack of age or injury severity stratification (Table 6).

**Table 6:** Literature review of fully thread screw fixation versus partial threaded cannulated screws for femoral neck fracture treatment

Authors	Year	Patients n	Patient age (years, range)	Classification and proportion (%)	Internal fixation^a^	Shortening	Complication (%)	Screw migration	Level of evidence
Chiang MH et al. [15]	2019	50	71.7 (37–95)	Pauwels I-II 45 (90%); Pauwels III 5 (10%)	17 by FTHCS; 33 by PTS	Significant shortening (> 5 mm) in both PTS (27.6%) and FTHCS (31.1%); no difference in length of neck shortening and neck-shaft angle tendency	1 nonunion and 2 ANFH in FTHCS (17.6%); 3 nonunion and 4 ANFH in PTS (21.1%)	N	Retrospective cohort study-III
Weil et al. [13]	2018	65	65.7 (14–91)	Garden I–II 59 (91%); Garden III–IV 6 (9%)	24 by FTCS; 41 by PTS	Smaller amounts of shortening with moderate or severe (> 5 mm) in FTCS; more valgus neck-shaft angle in PTS.	3 nonunion, 3 varus collapse and implant failure, 2 ANFH in FTCS (33.3%); 6 ANFH, 3 nonunion in PTS (22.0%)	17 screw pullout more than 5 mm in PTS; none in FTCS	Prospective case series with historical controls study-III
Zhang B et al. [10]	2018	59	50.2 (20–65)	Vertical femoral neck fracture (VN angle > 20°)	31 by PTS; 28 by FTHCS	9 shortening in PTS; 2 in FTHCS	7 nonunion in PTS; 1 in FTHCS; 13 fixation failure in PTS; 4 in FTHCS; 7 Varus deformity in PTS; 1 in FTHCS; 3 fracture displacement in PTS; 1 in FTHCS	10 nail withdrawal in PTS; 2 in FTHCS	Prospective comparative study-II
Okcu et al. [16]	2015	44	41.5 (21–70)	Pauwels I-II 21 (48%); Pauwels III 23(52%)	22 by 3 or 4 FTHCS; 22 by 3 or 4 PTS	N	4 nonunion and 4 varus malunion in FTHCS; 1 nonunion and 1 varus malunion in PTS	N	Prospective comparative study-II
Boraiah et al. [14]	2010	54	78 (48–100)	Garden I–II 25 (46%), Garden III–IV 29 (54%)	54 by FTCS coupled with either DHS or DHHS	Vector on the z-axis a linear displacement of 1.98mm. Change in screw-shaft angle 0.6°. Femoral neck offset 3.5 mm, abductor lever arm length 1.5 mm	2 nonunion failure and 1 ANFH; 7 residual greater trochanteric pain related to hardware	No screw pullout; average screw tip migration in *x*-, *y*- and, *z*- axis vector 0.7, 0.9, and 1.7 mm	Retrospective with historical controls-IV

*n* Number, *N* No mentioned, *FTHCS* Fully threaded headless cannulated screw, *PTS* Partial threaded cannulated screw, *FTCS* Fully threaded cannulated screw, *DHS* Dynamic hip screw, *DHHS* Dynamic helical hip screw

^a^All were treated with three parallel cancellous screws either FTCS or PTS

**Fig. 4:**
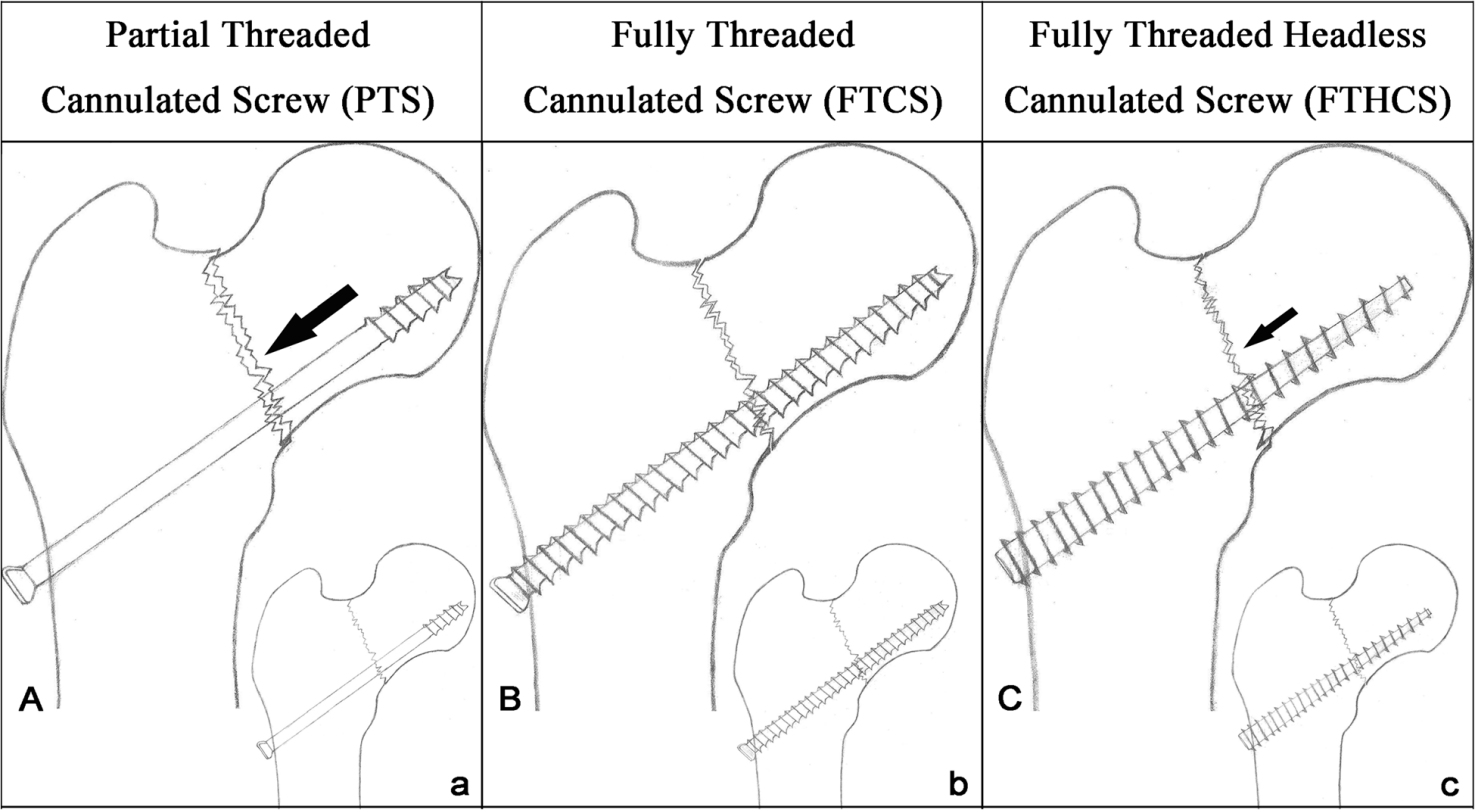
Schematic of three different cannulated screws used for fixation of FNF. The initial states of FNF fixed by (**a**) partial threaded cannulated screws (PTS), (**b**) fully threaded cannulated screw (FTCS), and (**c**) fully threaded headless cannulated screw (FTHCS) are represented in the lower right-hand portion of each figure. **a** There is a definite sliding mechanism during FNF healing in PTS fixation (large downward black arrow), which results in an observable dynamic compression across the fracture site. However, the proximal fracture fragment and PTS may move lateral-distally resulting in neck shortening and lateral screw protrusion, especially in comminuted fractures. **b** In contrast, the FTCS may prevent the femoral head from migrating along the screws, given the lack of a sliding mechanism. However, there may be a gap present at the fracture site 2–3 weeks postoperatively as a result of bone resorption or residual malreduction, particularly in comminuted fractures. **c** In FTHCS fixation, there is the possibility of an asymptotical sliding mechanism (small black arrow) due to the tapered profile of the screw. However, the fully threaded length results in length control structure, which may compromise the sliding efficacy during healing

The biomechanics of the FTCHS and PTS may provide insight into the clinical findings observed in our study. Two types of fully threaded screws have been used for FNF treatment, the fully threaded cannulated screw (FTCS) with normal head, cylindrical profile and equidistant pitch [13, 14], and the FTHCS. Although both are fully threaded, their fixation mechanisms are distinctly different. FTCS in FNF fixation was intended for use as a non-sliding, length-stable construct to prevent femoral neck shortening [14]. However, the FTCS lacks the sliding effect necessary for optimum healing and cannot function as a lag screw during implantation. As such, a gap may be present at the fracture site due to bone resorption or residual malreduction, particularly in comminuted fractures [26]. On the other hand, PTS function as sliding implants to provide dynamic compression during surgery and sliding during healing. However, the proximal fracture fragment and PTS may move lateral-distally, resulting in neck shortening and lateral screw protrusion, especially in comminuted fractures (Fig. 4).

The FTHCS, with a tapered profile and variable pitch [10, 15, 16, 25], functions somewhere in-between the FTCS and the PTS as an implant with static sliding or compression (asymptotical-sliding) and certain length stable properties (length-control construct) (Fig. 4). The sliding or compression between two fragments supplied by the FTHCS could be achieved by two plausible mechanisms. First, the tapered profile was designed to allow the threads to purchase new bone with each turn, gaining compression and maximizing pullout strength along its entire length. Second, the variable thread pitch was designed with wider thread at the tip of the screw, allowing faster bone penetration than the finer trailing threads and gradually compressing the two fragments as the screw is advanced. Because of these two mechanisms, the proximal fragment could also slide along the screw during fracture healing. At the same time, the full thread length design can better handle the cyclic loading that may occur during healing and function as a length control construct, similar to the FTCS.

One interesting finding in our study that deserves special attention is the medial screw migration seen in the FTHCS cohort. Fixation loosening in traditional PTS involves fracture compression along sliding implants during healing, which often leads to femoral neck shortening and/or lateral screw withdrawal resulting in greater trochanteric irritation by prominent lateral implants [11, 12]. However, an atypical mechanism of fixation loosening involving medial penetration of the FTHCS through the articular surface of femoral head was identified in our study. It is speculated that the screw configuration and geometry could be the most significant factor to explain this model of failure. First, the sharp threads at the end of the headless screw may lock with the lateral cortex of the proximal femur, making these two structures (screws and distal fragment) whole. A secondary factor is the possibility of the femoral head fragment (proximal fragment) easily sliding along the axis of the screw due to its tapered profile and variable thread pitch. Finally, the screw has a sharp tip with a relatively small cross-sectional surface with threads that are convex toward the subchondral bone, providing less resistance to medial migration. In fact, failure may not have been due to medial migration of the screw, but rather by the lateral sliding and collapse of the femoral head fragment. The above-mentioned factors in combination result in the medial screw protrusion, particularly in cases with posterior cortex communication. We observed that the inferior screw of the inverted triangle configuration appeared to the one to medially migrate. One interpretation may be that there were varying degrees of varus collapse of the femoral neck fragment due to less support of posterior-inferior cortex communication and bone absorption. As a result, for high-energy fracture pattern cases, we support the use of a regular triangle configuration by FTHCS fixation to form the “medial buttress” framework [10, 25].

The strengths of our study include the clinical relevance, the number of patients included in our analysis, and the discriminant analysis of the fixation complications based on fracture patterns. Other strengths include use of a regression model after controlling for important confounders in the cohort when comparing the effect of the implant selection.

Limitations in the present study design are outlined here. First, it was not a randomized trial and, therefore, it is possible that factors other than the choice of screw for fixation of FNF might have contributed to the observed differences between the groups after surgery. However, the present groups were very well matched on all characteristics, including variables relevant to surgical fixation, healing, and postoperative rehabilitation of FNF. The effect of changes in medical practice over time illustrates a principle confounding limitation of prospective cohort studies that utilize historic controls with the longer difference in time, the greater likelihood for confounders to bias the results [27]. However, practice patterns for treatment of FNF have not changed significantly at our institution from the time of the historical control treatment group to the end of the prospective cohort enrollment (January 2014–June 2017). Additionally, the close temporal recruitment of prospective patients with historical controls also gives us a certainty that our population is reflective of the type of patient with this injury, mitigating selection bias. Although the average follow-up time is sufficient to detect healing-related complications, it may be inadequate to detect ANFH. However, only a few of the patients who healed had early radiographic signs of ANFH without clinical manifestation.

## Conclusions

In high-energy FNF patterns in young adult patients, the rate of fixation failure is significantly lower with the use of FTHCS when compared with PTS, while there appears to be no difference in the rates of nonunion or ANFH. There was no significant difference in the rate of fixation failure, nonunion, or ANFH between FTHCS and PTS fixation in the low-energy fracture group. A distinctive medial migration fixation loosening mechanism was identified in the FTHCS cohort with high-energy FNF, thought to be multifactorial in etiology in the setting of lateral sliding and collapse of the femoral head fragment. The present study builds on the existing literature supporting the use of FTHCS for the treatment of high-energy FNF in young adult patients. Longer-term follow-up and increased availability of outcomes would further enhance the validity of this conclusion.

## Data Availability

Not applicable.

## Abbreviations

FNF: Femoral neck fractures
FTHCS: Fully threaded headless cannulated screws
FTCS: Fully threaded cannulated screw
PTS: Partial threaded cannulated screws
ANFH: Avascular necrosis of the femoral head
ISS: Injury severity scale
AP: Anteroposterior
CT: Computed tomography
MRI: Magnetic resonance imaging
VN: Vertical of the neck axis
OR: Odds ratios
CI: Confidence intervals
